# Another Stone in the Arch

**Published:** 1902-05

**Authors:** 


### EDITORIALS.

### ANOTHER STONE IN THE ARCH.

One by one are added the cut stones of legislation that soon will arch over the entire country with veterinary laws regulating, through State boards, the practice of veterinary science.

Much has been said in these columns, much has been done by the Journal and by members of its editorial staff in urging in the past a more united front in the State of New Jersey, and we have rejoiced in common with all others at the great results obtained there in establishing one single strong association. Following in the path of this great stride, New Jersey now adds additional safeguards to her vested interests in the animal industry; strengthens veterinary education all over the land; lifts her veterinary profession to a higher plane, and makes for those who represent the State in the veterinary profession a body of enthusiastic and successful organizers, workers, and association members to which, henceforth, all other associations must look to their own laurels lest they be dimmed in the bright light now reflected from New Jersey. We congratulate the profession in New Jersey on the securing a good law, the creation of a State board, the closing up of the arch of triumph in veterinary progress. To those who have led the way we offer special congratulations, and to those who will constitute her first board we extend our sincere wishes. The new law and the new board will be found elsewhere in this number, and the law and the board are assurances of a better state of affairs in New Jersey and a demand that the law will not only be respected, but its mandates obeyed.

